# The Hospitals Association

**Published:** 1889-06-08

**Authors:** 


### The Hospitals Association.

DR. BRISTOWE'S ADDRESS.

The fifth annual meeting of this association was held at the Westminster Town Hall on Wednesday, the 29th ult., the president, Dr. Bristowe, F.R.S., in the chair. Amongst those present were Sir William Moore, General Keatinge, Messrs. H. C. Burdett, Timothy Holmes, Surgeon-Major Ince, Colonel Montefiore, Dr. Potter, Dr. Hide, Richard B. Martin, Percy Wigram, P. J. Michelli, Thomas Ryan, and Howgrave Graham.

Mr. Ryan read the annual report, which we refer to elsewhere, the adoption of which was moved by the president.

Dr. Bristowe said : This time last year I felt it to be my duty, for reasons to which I need not now particularly refer, to inflict an address upon those members who honoured the annual meeting with their presence. No such imperative duty devolves upon me on the present occasion as I considered devolved upon me then. Yet it has appeared to me —and I believe my opinion is shared by at any rate some of the members of the council—that it is on the whole desirable that whoever is president of the association should inaugurate each year of his office with an address dealing with such matters of interest (whether already dealt with during the preceding session, or now under consideration, or simply in prospect), as seem to him suitable for the occasion or specially noteworthy.

On the whole I think we may look back upon the last session with considerable satisfaction. At most of our meetings we have had papers and discussions of much interest and value on subjects which are specially suitable for consideration by The Hospitals Association. Our meetings have been held largely at metropolitan hospitals, with the sanction of the authorities of these hospitals, and under the presidency either of one of the governors or of some member of the professional staff. And, as will be seen by the list of our proposed members, the council has been greatly strengthened by the addition to it of several of the treasurers of our large hospitals, and of other gentlemen who are interested in the medical welfare of the sick poor.

Of the important group of papers contributed during the last two sessions by Mr. Burdett Coutts, Mr. Nelson Hardy, Dr. Gilbart Smith, and Sir Sydney Waterlow on the difficult subject of raising adequate funds for the maintenance of our subscription hospitals, I do not propose to speak at any length. The papers may all be taken as the expression of the well-considered views of able men, who are entitled from long experience to hold opinions on the subject on which they have written. But they have treated it from different points of view, and the opinions expressed and the conclusions come to seem, at first sight, widely divergent, if not irreconcilable. This result is only what was foreseen. The association, in inviting papers upon the subject, applied of set purpose to gentlemen whose views were known or suspected to be at variance, in the hope that their papers might form the basis of an enquiry into the whole subject, and that out of their apparently conflicting views some satisfactory solution of the question might hereafter be evolved. I still trust that the association will be able to appoint a competent committee to enquire into and report on the whole subject.

Mr. Michelli's paper on "Hospital Extravagance and Expenditure" has clearly a close relation to the group of papers above referred to, inasmuch as legitimate reduction of hospital expenses has, so far as the essential duties of hospitals are concerned, the same effect as equivalent increase of subscriptions. And it deserves special attention, as coming from a man who has had large experience as secretary in two large metropolitan hospitals, and who has evidently bestowed much thought on the subject. With a great deal that Mr. Michelli says I entirely agree. He considers hospital extravagance under two heads, namely, Direct Extravagance and Indirect Extravagance. In the first category he places the waste connected with medical sundries and extras, and that occurring in the domestic department ; in the second category he includes the cost of printing, stationery, etc., advertising, and collection. There is no doubt whatever that there is apt to be, and often is, much extravagance and waste in regard to so-called medical sundries and extras, including under these terms diet. And it is difficult to know how to deal with the matter otherwise than by constant and close supervision by those in authority. The medical men must be the judges of what the patients under their charge need in the way of food, stimulants, medicines, and appliances ; and I take it that no treasurer or committee would venture to assume the responsibility of interfering in any ordinary case with a physician's or surgeon's freedom of action in such matters. At the same time there is a general tendency on the part of medical attendants at hospitals, thinking only of the actual interests of the patients under their charge, to order extras of all kinds without due regard to economy, and having ordered them to continue their use needlessly long. And this laxity of action on the part of the seniors involves a much greater laxity in the same sense on the part of the house officers who are acting under them and in their absence. I recollect, some years ago, that an assistant medical officer, without due thought, ordered for an out-patient a fortnight's supply of some special and newly-introduced medicine, and was inclined to stand upon his dignity and to insist upon his order being obeyed, although the apothecary showed him that the actual cost of the drug would amount to three or four pounds, and its exhibition was, to a large extent, only experimental. I repeat, however, that the lay managers of hospitals can rarely interfere in respect of the treatment of individual cases, but they may quite properly from time to time call the attention of the medical officers to the amount of extras of all kinds, for the ordering of which they are severally responsible. That has been the practice for years at St. Thomas's, and has certainly been effective to some extent in restraining the growth of expenditure on such matters. As to waste occurring in the domestic department, I have little personal knowledge. Nevertheless, I could recall a few very remarkable incidents proving such waste, were it necessary for me to do so. It is obvious, however, that in this department there are many opportunities for wastefulness, and that unless there be close and constant supervision, more or less serious waste is sure to arise. But it is what Mr. Mitchelli terms indirect extravagance which, as he states, and amply proves, is mainly answerable for waste of hospital funds. This form of extravagance belongs almost exclusively to hospitals supported by voluntary contributions, and is incurred mainly in obtaining subscriptions. And, indeed, he tells us that in many instances from 25 to 50 per cent., and even more, is thus expended, while "those who subscribe their money are in utter ignorance that only ten shillings of the sovereign reaches the poor for whom it is intended." He quotes figures to show that in the case of one hospital nearly five pounds per occupied bed are spent in advertising alone ; and that in that of another, over five pounds per occupied bed are spent in printing, stationery, and postage ; and he points out further that, though in the case of large medical charities, collectors and canvassers are as a rule paid only from 2½ to 5 per cent. commission, in some of the smaller and special hospitals there seems to be no limit to the commission which may be given. He was informed by a collector to several charities that he had been offered no less than

40 per cent. on new money if he would canvass for a particular small special hospital.

The concluding part of Mr. Michelli's paper is devoted to the subject of hospital accounts. He states that not only are the annual accounts which are published framed on principles which are irreconcilable with one another, but that also in large proportion the items of receipts and expenditure are so manipulated that even a skilled accountant finds them largely unintelligible, and that, therefore, any useful comparison between the financial managements of different institutions is rendered practically impossible. The Hospitals Association, from its foundation, has urged the keeping of hospital accounts on some uniform and intelligible plan, and it is sincerely to be hoped that before long this urgent reform, so important to the charitable public, and so necessary in the interests of all well-conducted institutions, will be carried into effect.

Mr. Michelli's paper has, if I recollect right, been rather severely handled, and been spoken of as though its effect, if not its aim, must be to damage hospitals. He has certainly not been mealy-mouthed, and I suspect that some of the adverse criticism has been due rather to his manner than to his matter. His paper seems to me both able and opportune, and I fail to see how the truth, which he tells, can be detrimental to the usefulness or prosperity of well-conducted and honest institutions, as I am proud to feel that all our larger hospitals, and many of our small ones, certainly are.

The most important paper of the session was undoubtedly that of Mr. Ryan, secretary to St. Mary's Hospital. As he tells us, the question of the conveyance of injured persons to the metropolitan hospitals was considered by the council of the association, and he was requested to prepare a paper on the subject with the object of bringing the matter before the association and the public, and of having it fully discussed. No better choice could have been made. The subject is one with which Mr. Ryan was well acquainted, and in which he was deeply interested, but it is obvious that for the purposes of his paper he took infinite pains to ascertain the exact truth with respect to existing arrangements in the metropolis, and to formulate a feasible scheme for perfecting them.

Mr. Ryan points out: 1st. That the existing ambulance arrangements for the removal of infectious cases to the hospitals of the Metropolitan Asylums Board are admirable; that there are three ambulance stations, which are situated adjoining the Eastern, South-Eastern, and Western Hospitals respectively, and also three ambulance steamboats for the transport of convalescing patients to the hospital at Darenth; that the stations are supplied with from 50 to 60 ambulance carriages of the pattern designed by Mr. John Furley; and that the practical working of the organisation is the admiration of everyone who has had the opportunity of witnessing it; and 2nd, that excellent provision is made by the St. John's Ambulance Association by their Invalid Transport Corps, under the management of Mr. Furley, for the removal of sick persons who are able to pay a small sum for their removal.

But, on the other hand, he shows that absolutely no provision exists for the removal of infectious cases to places other than the Asylums Board hospitals; that, as a rule, the arrangements for the removal of the sick poor to the workhouse infirmaries are exceedingly defective; and that the only systematised provision now existing for the removal of accidents is that administered by the metropolitan police and the City police.

As regards the removal of infectious cases to places other than the Asylums Board hospitals, he evidently approves, as all persons I think must who give any consideration to the matter, of the recommendation of the Ambulance Committee of the Metropolitan Asylums Board, to the effect that the Board should be empowered to use their ambulances for the removal of all classes suffering from infectious disorders. Up to the present time, so far as I know, no effect has been given by the Local Government Board to this recommendation.

As regards the removal of the sick to the workhouse infirmaries, he shows that, if the same provision were to be made at all infirmaries as is now made at a few of them, the arrangements in the metropolis for the removal of the sick poor would be practically perfect.

The main object of Mr. Ryan's paper, however, is the consideration of the question of the conveyance of persons injured or stricken with sudden illness in the streets, etc., to the various hospitals. As to this, he shows clearly, what all hospital authorities know only too well, that the existing arrangements in the metropolis (outside the City of London) are altogether inadequate, and I am afraid one must add discreditable. Within the metropolitan circle of four miles radius, including fifty square miles, there are forty-nine police ambulance stations, but these are very irregularly distributed, and in a certain area of ten square miles there are only five of them. Moreover, the ambulances in use are "cumbersome, rickety, antediluvian concerns, which ought to be promptly abolished." He calculates that under present conditions an ambulance could scarcely under the best of circumstances reach a case of accident under twenty minutes. Often the time would be much longer; and, as we know, many of the most serious accidents are, mainly in consequence of the difficulty of getting ambulances, conveyed in cabs or other equally inappropriate public vehicles.

If Mr. Ryan's paper had only demonstrated the utter inadequacy of present ambulance arrangements for the conveyance of medical and surgical cases of sudden emergency, its value would have been considerable. But Mr. Ryan has gone much further; for, as the result of careful and extensive investigation of the subject and much thought, he has shown how an ambulance service for such cases, perfectly efficient and worthy of the metropolis, could be instituted and carried out at a very moderate cost. He proposes as follows: That ambulance stations should be largely multiplied over the metropolis, that they should be provided not only at the police-stations, but also at the fire-brigade stations, at railway stations, and at hospitals, and, if possible, in other localities; that an ambulance litter and necessary accessories should be kept in each station; that negotiations should be entered into with the various authorities concerned in order to obtain, free of charge, standing-room in the premises indicated; that an appeal be made to the public for funds for the purchase and maintenance of ambulances; that the conduct of the negotiations, and of the operations necessary for raising the required funds, be undertaken by The Hospitals Association; and that the association appoint a sectional committee to transact the business and to manage the affairs of the ambulance service.

I cannot help congratulating the public generally, The Hospitals Association, and Mr. Ryan himself, on the fact that his proposals appear to be in process of early realisation. An influential and representative committee has been appointed by the council; the subscription list has been started with the munificent donation of £1,500; the form of ambulance to be employed has, I believe, been selected; and the committee propose to proceed, I hear, at once to order a sufficient number of litters to enable them to place one within each area of five hundred yards diameter comprised within the metropolitan circle of four miles radius.

There can be no doubt that this scheme will be carried out within a short time, and that London will thus be provided with what has long been sorely needed—namely, a sufficient and efficient street ambulance organisation.

It is, I think, a matter of legitimate pride to The Hospitals Association that within the last two years they have been the means of inaugurating two great public philanthropic measures—one, the National Pension Fund for Nurses, the design of which originated in the fertile and philanthropic brain of Mr. Burdett, the carrying out of which was made possible by the munificence of Lord Rothschild and Messrs. Gibbs, Hambro and Junius S. Morgan, and which is already successful beyond our most sanguine hopes; the other, the ambulance scheme for persons injured or stricken with sudden illness, of which I have just spoken at length, and which is in process of realisation.

The measures just referred to bring The Hospitals Association into close and beneficial relations with the metropolitan hospitals. This is as it should be, for The Hospitals Association (as its name implies) was always intended to be an association—partly, no doubt, of persons only interested in the work of hospitals, but mainly of the governors, medical staffs, secretaries, matrons, and other persons engaged officially in hospital duties. For this reason, also, I am glad that, as I have already mentioned, most of our sessional meetings of the last year have taken place under the auspices of the authorities in the board-rooms of the large metropolitan hospitals. While thanking the treasurers and governors of these institutions for their kindness to the association, I may be permitted to hope that this kindness will be continued.

In conclusion, I must repeat my congratulations to the association on the addition to its council of so many men of eminence who, from their position, are specially suitable and welcome members. It is a proof that the work of the association has their sympathies; and it is an encouragement and a guarantee for the performance of good work in the future. I hope I may be excused for emphasising the fact that on the council we have now the treasurer of St. Bartholomew's, Sir Sydney Waterlow; the treasurer of St. Thomas s, Alderman Stone; the treasurer of Guy's, Mr. Lushington; and the chairman of the London Hospital, Mr. Carr-Gomm; and for thanking, on behalf of the association, the Most Honourable the Marquis of Carmarthen, not only for joining the council, but for consenting to occupy the post of chairman of council."

Amongst other speakers was Mr. Richard B. Martin, joint hon. secretary of the Hospital Sunday Fund, who expressed his sense of the usefulness of the association, and his gratification that it was now so firmly established and admirably organised.

Mr. Burdett pointed out that everyone interested in a hospital would be able to apply to the association with the assured confidence that any information they required would be immediately forthcoming, seeing that the two hon. secretaries—Messrs. Michelli and Ryan—were amongst the most intelligent and most experienced of hospital secretaries. He paid a tribute of respect to the memory of the late Major Ross, M.P., whose loss would long be felt in the hospital world, and congratulated the association that Lord Carmarthen, M.P., had accepted the chairmanship of the council.

Dr. Steele congratulated the association that the Nurses Pension Fund was now an accomplished fact, and that the street Ambulance branch had been successfully organised.

Surgeon-Major Ince, General Keatinge, the Rev. Canon Marshall, and Sir William Moore also spoke.

At the close of the proceedings the president, Dr. Bristowe, stated that his work in connection with the Association throughout the year had been entirely pleasant, as it had lived down the attacks made upon it, and its position as an association of great public utility was now assured.

